# Idiopathic Recurrent Serositis: A Multispecialty Challenge Resolved With Colchicine

**DOI:** 10.7759/cureus.95436

**Published:** 2025-10-26

**Authors:** Shivashankari Dakshinamoorthy, Renuka Renuka, Irfan Tariq, Deepak Albana

**Affiliations:** 1 Acute Medicine, University Hospitals Birmingham NHS Foundation Trust, Birmingham, GBR; 2 Diabetes and Endocrinology, University Hospitals Birmingham NHS Foundation Trust, Birmingham, GBR; 3 Geriatrics, University Hospitals Birmingham NHS Foundation Trust, Birmingham, GBR

**Keywords:** colchicine treatment, complex pleural effusion, diagnostic efficacy, gross ascites, serositis

## Abstract

Idiopathic recurrent serositis (IRS) is a rare and diagnostically challenging condition characterised by recurrent inflammation of serous membranes, occurring without any identifiable infection, malignancy, or autoimmune cause. We present the case of a 32-year-old female who was initially admitted with dyspnoea and a right pleural effusion. Pleural fluid analysis revealed an exudate with negative microbiology, cytology, and tuberculosis testing, and she was treated empirically with antibiotics. Over the following months, the patient developed recurrent symptoms, including progressive abdominal distension due to gross ascites and a small pericardial effusion. An extensive multidisciplinary evaluation involving infectious disease, gynecology, respiratory medicine, and rheumatology yielded no definitive findings. Cross-sectional imaging and positron emission tomography (PET) excluded malignancy, while pleural and peritoneal biopsies demonstrated nonspecific chronic inflammation without granulomas, infection, or malignant cells. Extensive autoimmune, viral, and mycobacterial serological panels were negative. Intermittent cultures grew environmental organisms considered contaminants, and broad-spectrum antimicrobials did not alter the clinical course.

Following referral to a national amyloidosis and autoinflammatory disease service, a diagnosis of IRS was suggested. The patient was started on colchicine, titrated from 500 mcg to 1.5-2 mg daily, alongside nonsteroidal anti-inflammatory drugs (NSAIDs) for breakthrough pain, resulting in marked clinical improvement, resolution of effusions, and sustained remission at follow-up, with return to normal activities. This report highlights the diagnostic complexity of recurrent serositis, where nonspecific clinical features and broad differentials often lead to extensive investigations and delays in effective treatment. Colchicine, through its anti-inflammatory effects on neutrophil function, provided durable control in this case, consistent with its established efficacy in familial Mediterranean fever and other autoinflammatory conditions. Clinicians should consider IRS in patients with unexplained recurrent effusions, and colchicine should be recognised as an effective first-line therapeutic option once secondary causes are excluded.

## Introduction

Recurrent serositis, encompassing relapsing pleuritis, pericarditis, or ascites, is a significant diagnostic and therapeutic challenge. As reported by Adler et al., recurrence is particularly well described in pericarditis, affecting 15-30% of patients after an initial episode, with some cohorts reporting rates up to 50% [1]. Its pathophysiology is heterogeneous, which includes infection, malignancy, autoimmune-complex disease, and autoinflammatory syndromes [2]. Familial Mediterranean fever (FMF) and tumour necrosis factor receptor-associated periodic syndrome (TRAPS) illustrate the role of Interleukin-1 (IL-1)-mediated innate immune dysregulation [3,4]. Recent mechanistic insights further substantiate the link between psychological or physiological stress and autoinflammatory flares. Skendros et al. demonstrated that stress-induced upregulation of regulated in development and DNA damage responses 1 (REDD1) activates IL-1β-mediated inflammatory pathways in FMF, promoting autophagy-driven neutrophil extracellular trap formation and precipitating attacks [3]. This provides a biological basis for the observed association between emotional stress and clinical relapses in FMF and related autoinflammatory syndromes.

Diagnosis follows a stepwise approach involving fluid analysis, cytology and cultures, imaging studies, and autoimmune serological testing. As noted by Caterson et al., the diagnosis of autoinflammatory syndromes has become increasingly individualised with the growing accessibility of gene panel testing. [5]. Historically, only a limited number of syndromic diseases were recognised; however, this number has expanded in recent years. The ability to test every suspected case remains limited in most hospitals, with specialist centres being far and few [5]. The main concern, however, is the lack of testing sensitivity. Pleural cytology has a mean sensitivity of ~60% for malignancy detection. Typically, the metastatic carcinoma and TB diagnostics may be inconclusive, necessitating repeat sampling or biopsy when suspicion persists [6,7]. Persistent barriers include delayed recognition, restricted test sensitivity, and limited global access to biologics [5].

Although combined therapy with colchicine and nonsteroidal anti-inflammatory drugs (NSAIDs) is the first-line treatment for Idiopathic recurrent serositis (IRS), previous studies have indicated that colchicine monotherapy may serve as an effective alternative to lower the risk of recurrence [4,6]. In this context, we present a unique case of a patient who, after months of recurrent symptoms and multiple specialist evaluations, was ultimately diagnosed with an autoinflammatory syndrome that responded well to colchicine therapy.

## Case presentation

**Figure 1:**
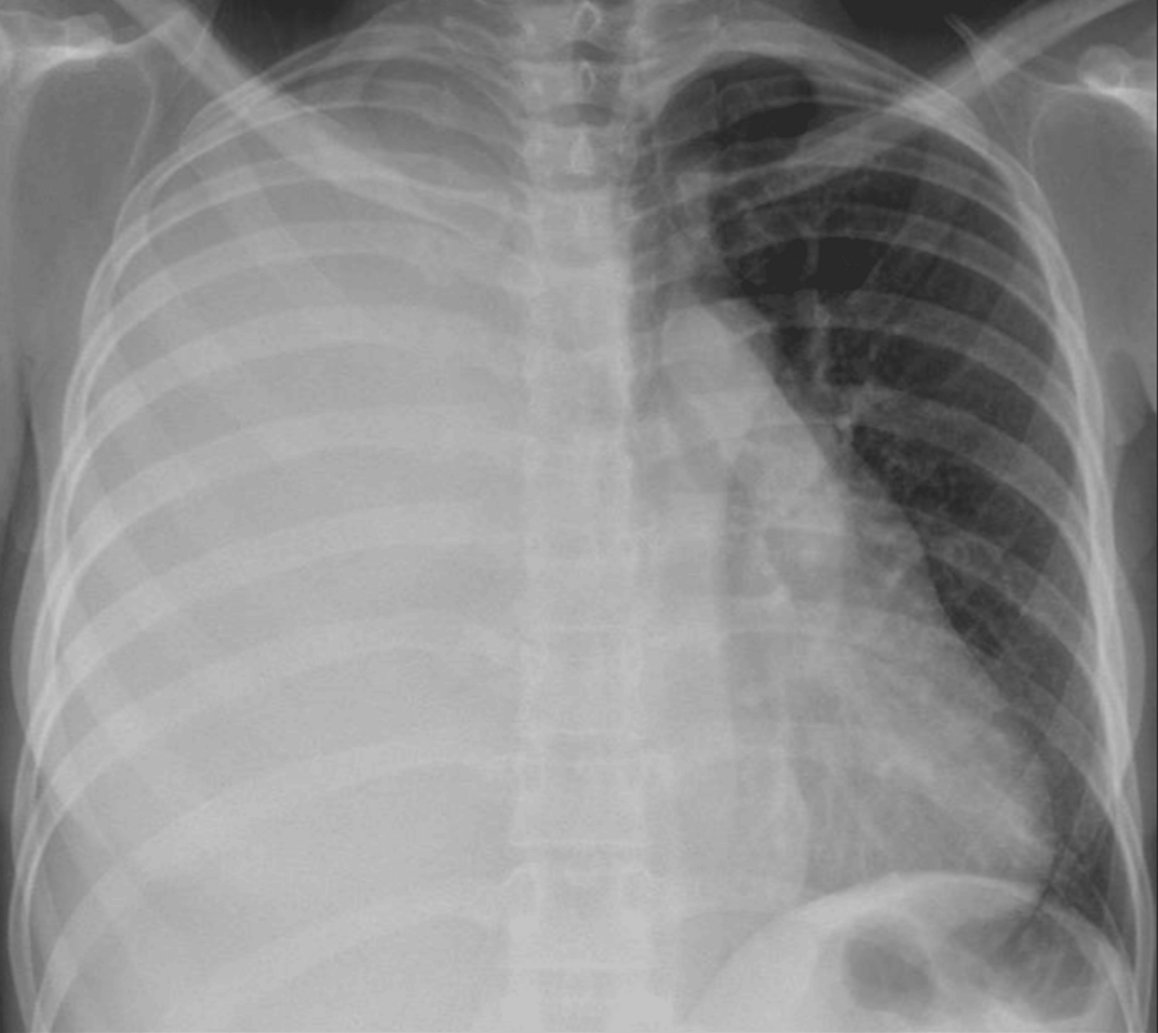
Chest X-ray on first presentation revealing a massive right-sided pleural effusion resulting in complete opacification of the right hemithorax and leftward mediastinal shift

A 32-year-old female with no significant past medical history presented with progressive shortness of breath. She had experienced similar symptoms one year earlier while in Nigeria, where she was empirically treated for a lower respiratory tract infection. Discharge notes from that evaluation provided limited clinical details, noting only a diagnosis of a small pleural effusion, likely secondary to infection. During her admission under our care, the initial chest X-ray showed a massive right-sided pleural effusion (Figure 1), which was aspirated. Investigations of pleural fluid, including microbiology, did not reveal any organisms or growth. The biochemistry findings had confirmed the exudative nature of the fluid, and cytology revealed no malignant cells on the aspirate. TB analysis of the fluid culture and polymerase chain reaction (PCR) tests were negative. Ziehl-Neelsen staining was negative for acid-fast bacilli. She was discharged on antibiotic therapy with a plan to repeat a chest X-ray in six weeks, which reportedly returned normal. She was also scheduled for follow-up at the chest clinic under the care of the Respiratory team.

**Figure 2:**
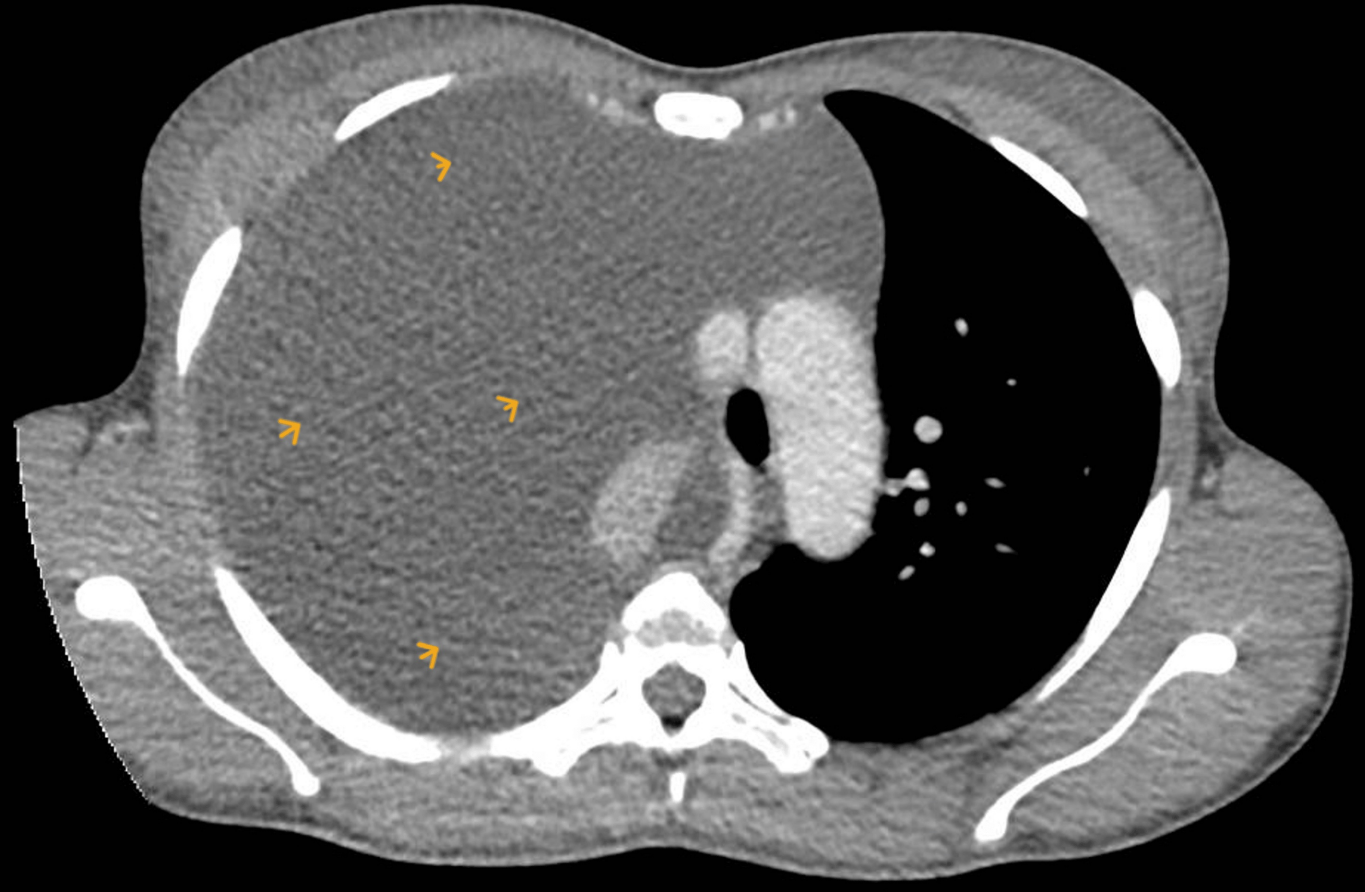
CT scan showing a large volume right-sided pleural effusion (yellow arrows) CT: computed tomography

**Figure 3:**
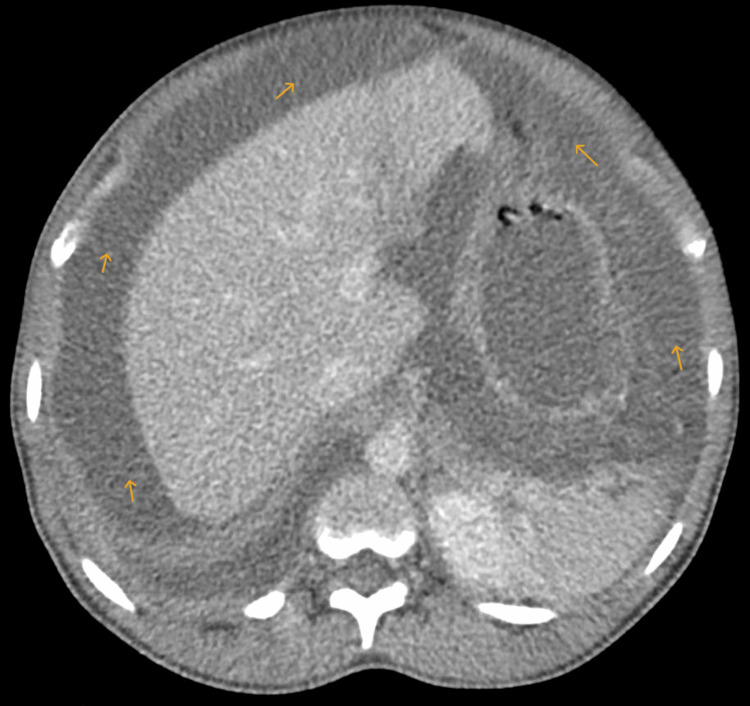
CT scan showing accumulation of ascitic fluid in the peritoneal cavity (yellow arrows) CT: computed tomography

Eight months later, the patient re-presented with abdominal distension, discomfort, and shifting dullness. She was afebrile and had no orogenital lesions, rashes, or any visual symptoms or signs. Blood tests showed an elevated C-reactive protein (126 mg/L) with otherwise normal renal function and full blood count. Contrast-enhanced CT of the thorax, abdomen, and pelvis (Figures 2, 3) demonstrated the recurrence of a massive right pleural effusion resulting in near complete collapse of the right lung and mediastinal shift, in addition to gross ascites, bulky uterine fibroids, and a left adnexal soft tissue mass with a cystic component.

Extensive serological testing, including antinuclear antibodies (ANA), antineutrophil cytoplasmic antibodies (ANCA), anti-liver/kidney microsomal (anti-LKM), anti-smooth muscle antibody (ASMA), antimitochondrial antibody (AMA), complement levels, dsDNA, immunoglobulins, electrophoresis, HIV, hepatitis, and extended viral panels, was unremarkable (Table 1). A subsequent positron emission tomography-computed tomography (PET-CT) scan showed no evidence of metabolically active malignancy, infection, or any structural cause for the ascites or pleural effusion. However, moderate uptake was noted in residual thymic tissue, likely representing benign thymic hyperplasia. Overall, these findings raised the possibility of an underlying connective tissue disorder

**Table 1:** Summary of laboratory, imaging, and pathological investigations Table outlining the results of the patient’s investigations, including hematological, biochemical, autoimmune, infectious disease screening, fluid analyses, imaging findings, biopsy reports, and genetic testing. Reference ranges are provided where applicable for the clinical context WBC: white blood cell count; CRP: C-reactive protein; LDH: lactate dehydrogenase; ANA: antinuclear antibody; ANCA: antineutrophil cytoplasmic antibody; LKM: liver kidney microsomal antibody; ASMA: anti-smooth muscle antibody; AMA: antimitochondrial antibody; dsDNA: double-stranded DNA; Ig, immunoglobulin; SAAG: serum–ascites albumin gradient; TB: tuberculosis; PCR: polymerase chain reaction; EF: ejection fraction; VATS: video-assisted thoracoscopic surgery; CT TAP: computed tomography thorax, abdomen, and pelvis; PET-CT: positron emission tomography-computed tomography

Investigation	Result	Reference value
Full blood count - WBC	6.75 (normal)	3.00-10.90 x 10^9^/L
CRP	126 (high)	0-5 mg/L
Serum lactate dehydrogenase	249 (slightly raised)	125-220 U/L
Serum protein	71 (normal)	60-80 /L
Autoimmune analysis		
ANA	Negative	
ANCA	Negative	
LKM antibody	Negative	
ASMA	Negative	
AMA	Negative	
Mitochondrial antibody	Negative	
Smooth muscle antibody	Negative	
Immunoglobulin G	13.85	6.00-16.00 g/L
Immunoglobulin A	1.78	0.80-2.80 g/L
Immunoglobulin M	1.5	0.50-1.90 g/L
dsDNA antibody	<0.6	0.6-9.9 IU/ml
IgG cardiolipin antibody	0.9	0.5-23.8 GPLU/ml
IgM cardiolipin antibody	2.8	0.9-29.9 MPLU/ml
Beta2 glycoprotein G	1	0.8-17.7 U/ml
Beta2 glycoprotein M	<2.4	2.4-5.7 U/ml
Complement C3	1.58	0.75-1.65 g/L
Complement C4	0.47	0.14-0.54 g/L
Electrophoresis	No paraprotein band seen	
Infectious diseases screen		
HIV 1 and 2 antigen/antibody	Negative	
Hepatitis B HBsAg	Negative	
Hepatitis C antibody	Negative	
T-SPOT TB	Negative	
SARS-CoV-2 RNA	Negative	
Respiratory syncytial virus RNA	Negative	
Influenza A virus RNA	Negative	
Influenza B virus RNA	Negative	
Parainfluenza virus RNA	Negative	
Rhinovirus RNA	Negative	
Human metapneumovirus RNA	Negative	
Adenovirus DNA	Negative	
Blood cultures	Negative	
Pleural fluid - exudate	Yes	
Pleural fluid - cytology	Normal cytology	
Pleural fluid - microbiology	Acinetobacter species (environmental contaminant)	
Ascitic fluid - microbiology	No organisms	
Fluid albumin	21	>11 g/L (depends on SAAG)
Fluid LDH	726 (high)	225–300 U/L
Fluid total protein	42 (high)	<25 g/L (transudate)/>25 g/L (exudate)
Microscopy	Mild prominence of lymphocytes. No malignant cells seen	
SAAG	0.5	<1.1 g/dL (exudative type)
TB analysis		
IGRA testing	Negative	
Sputum cultures	Negative	
Acid-fast bacilli	Negative	
TB-PCR	Negative	
Mycobacteria	Negative	
Alpha fetoprotein	<1.7	<7.4 ku/L
Imaging analysis		
CT TAP with contrast	Right-sided pleural effusion, gross ascites, bulky uterine fibroids, adnexal mass	
PET CT scan	No evidence of malignancy/infection/structural cause for effusions	
Echocardiography	Small pericardial effusion, EF 60%	
Peritoneal biopsy	No granulomatous inflammation. No TB or malignancy	
VATS	Scar tissue, no atypia or malignancy	
R413 gene analysis and amyloidosis testing	Negative	

The pleural fluid profile and cytological findings were identical to those observed during the prior presentation, showing no new diagnostic features. Ascitic fluid analyses confirmed an exudative effusion (serum-ascites albumin gradient (SAAG) <1.1) with lymphocyte predominance and negative cytology. Fluid cultures grew *Acinetobacter baumannii* and *Mycobacterium chimaera*, both of which are considered environmental contaminants. Tuberculosis PCR and interferon-gamma release assay (IGRA) were negative. Gynaecological evaluation attributed pelvic findings to benign fibroids, unrelated to the patient's presentation.

Video-assisted thoracoscopic surgery (VATS) pleural biopsies demonstrated nonspecific chronic inflammation without evidence of malignancy or infection. Broad-spectrum antibiotics (clarithromycin and ciprofloxacin) were administered after *Pseudomonas *was isolated from chest drain fluid, although repeat VATS biopsy remained negative on histology and cultures. Despite the persistence of pleural effusion and ascites, the patient remained clinically stable with no systemic symptoms. Her only concerns were abdominal distension causing discomfort and a sense of generalised fatigue. She remained haemodynamically stable. Peritoneal biopsies revealed no malignant cells, granulomatous inflammation, or mycobacterial infection with negative PCR and cultures for tuberculosis.

After a multidisciplinary review, referral to a national amyloidosis and autoinflammatory disease service in London led to a working diagnosis of IRS, and genetic testing for a relevant autoinflammatory mutation was initiated. R413 autoinflammatory disorders testing was negative, alongside a negative screen for hereditary amyloidosis testing.

**Figure 4:**
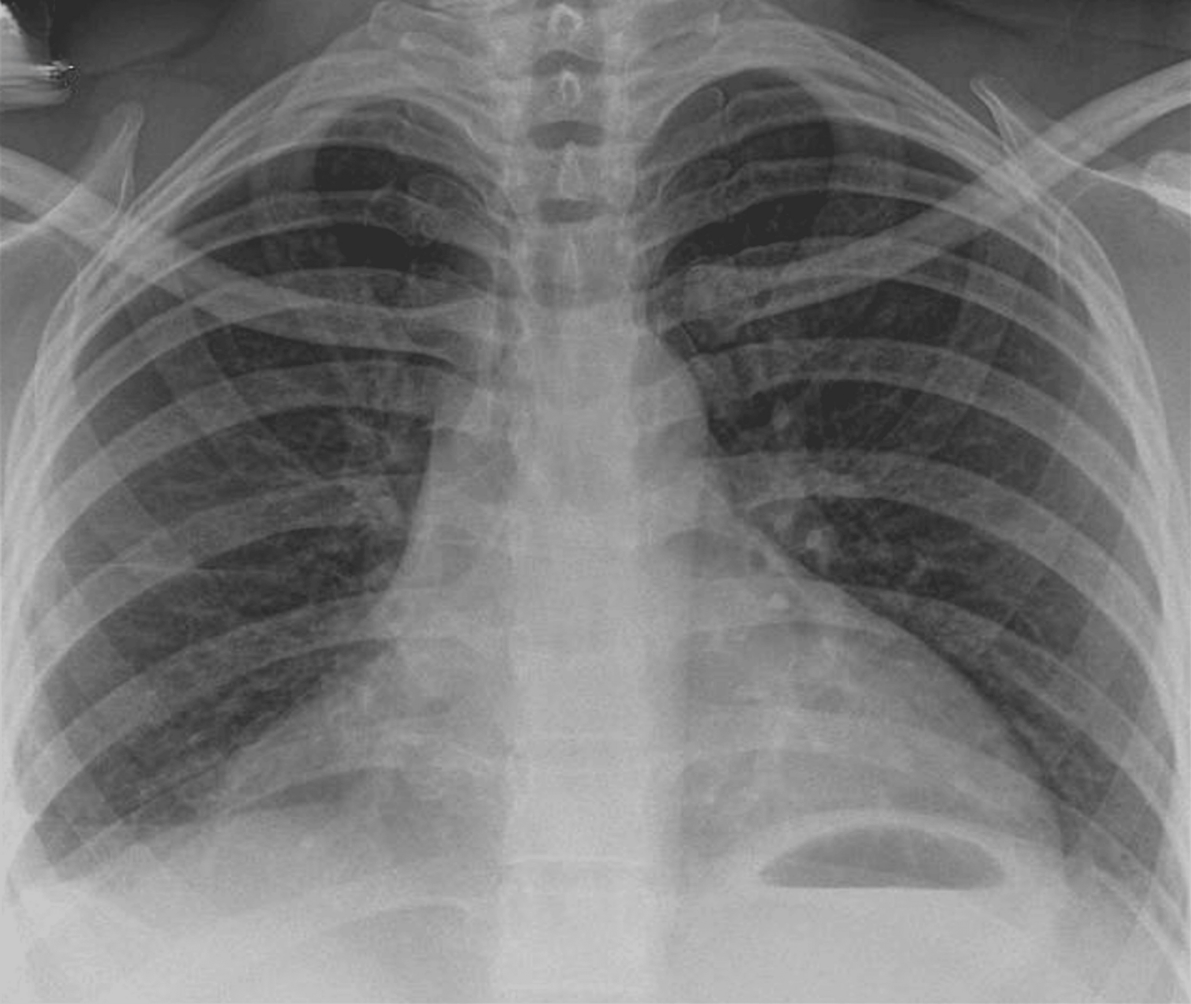
Chest X-ray showing resolution of pleural effusion following colchicine therapy

Colchicine prophylaxis was started at a dose of 500 mcg daily and gradually increased to 1.5-2.0 mg. For acute episodes, ibuprofen 200 mg was prescribed, in line with the approach described by Roy et al. [6]. The patient achieved marked clinical improvement, with complete resolution of ascites and pleural effusion (Figure 4), and has remained symptom-free in sustained remission at follow-up, having returned to her full daily activities.

## Discussion

IRS is characterised by inflammation of the serous membranes without an identifiable underlying trigger [6]. It is generally considered autoinflammatory, arising from dysregulated innate immune responses. The condition presents a significant diagnostic challenge due to nonspecific clinical features and a broad differential, necessitating the exclusion of infectious, malignant, and autoimmune causes [2,4]. In our patient, extensive investigations including imaging, fluid analyses, biopsies, and comprehensive serological testing ruled out secondary aetiologies. As previously reported by Roy et al., persistently elevated inflammatory markers alongside recurrent pleural, pericardial, and peritoneal effusions in a haemodynamically stable patient pointed towards a non-infective, inflammatory process [6].

We conducted a literature review using the databases MEDLINE, Embase, and PubMed to examine the aetiology of serositis and its association with autoinflammatory syndromes. Among more than 20 well-characterised seronegative conditions, serositis is a common feature, occurring in disorders such as FMF, TRAPS, and the rarer adenosine deaminase 2 deficiency (DADA2) and NLRP2 spectrum disorders [3,8]. As reported by Rech et al., the average time to diagnosis was three years in children and adolescents, compared to 14 years in adults [8]. Before the correct diagnosis of an autoinflammatory disease was established, many patients had received multiple misdiagnoses, including psychosomatic disorders [2]. Management typically involves corticosteroids or colchicine, often combined with conventional synthetic disease-modifying anti-rheumatic drugs (csDMARDs) and biologics such as adalimumab, anakinra, or tocilizumab [9]. In navigating treatment options for rare rheumatic diseases, clinicians frequently rely on existing literature and available case reports.

In our case, colchicine was initiated and titrated to 1.5-2 mg daily, alongside NSAIDs for acute episodes, resulting in marked clinical improvement and sustained remission [6]. Steroids may be employed to reduce inflammation and prevent effusions; however, prolonged use can lead to substantial side effects. In contrast, colchicine and NSAIDs have proven effective in this case, leading to sustained resolution of symptoms and signs to date. Massaro et al. previously demonstrated that colchicine effectively reduces inflammation, prevents fluid accumulation, and minimises relapses, while avoiding the long-term adverse effects linked to corticosteroid use [4]. Our findings align with the existing literature, supporting the use of colchicine as a first-line therapeutic option in recurrent serositis, even in cases where the genetic confirmation of an autoinflammatory syndrome is unavailable [4-6]. Early consideration of colchicine in patients with unexplained recurrent serositis may reduce unnecessary investigations, shorten the diagnostic journey, and improve outcomes.

## Conclusions

IRS poses a considerable diagnostic challenge because of its nonspecific clinical features and wide-ranging differential diagnoses. In addition to excluding infections - both common and rare, such as pneumonia, HIV, and TB - clinicians must remain vigilant to avoid missing an underlying malignancy. A focused history and examination, including travel history, gynaecological history (where relevant), and drug use, is also vital to refine differential diagnoses. This case of recurrent serositis illustrates an inflammatory phenotype where resolution of symptoms occurred only after pharmacological intervention. Such cases are rare and unique and often pose a challenge in establishing a specific diagnosis. Importantly, autoinflammatory and recurrent inflammatory conditions should remain part of the differential when no clear cause is identified. In cases where IRS is considered, colchicine may represent an effective first-line therapeutic option, offering sustained symptom resolution and prevention of relapse. Early initiation of colchicine could therefore improve patient outcomes in similar scenarios.
